# Association between delay in intensive care unit admission and the host response in patients with community-acquired pneumonia

**DOI:** 10.1186/s13613-021-00930-5

**Published:** 2021-09-28

**Authors:** Liza Pereverzeva, Fabrice Uhel, Hessel Peters Sengers, Olaf L. Cremer, Marcus J. Schultz, Marc M. J. Bonten, Brendon P. Scicluna, Tom van der Poll, the MARS consortium

**Affiliations:** 1 Center for Experimental and Molecular Medicine, Amsterdam University Medical Centers, Location Academic Medical Center, University of Amsterdam, Meibergdreef 9, G2-129, 1105 AZ Amsterdam, The Netherlands; 2 Department of Intensive Care Medicine, University Medical Center Utrecht, Utrecht, The Netherlands; 3 Department of Intensive Care, Amsterdam University Medical Centers, Location Academic Medical Center, University of Amsterdam, Amsterdam, The Netherlands; 4 Mahidol‑Oxford Tropical Medicine Research Unit (MORU), Mahidol University, Bangkok, Thailand; 5 Nuffield Department of Medicine, University of Oxford, Oxford, UK; 6 Department of Medical Microbiology, University Medical Center Utrecht, Utrecht, The Netherlands; 7 Julius Center for Health Sciences and Primary Care, University Medical Center Utrecht, Utrecht, The Netherlands; 8 Department of Clinical Epidemiology, Biostatistics, and Bioinformatics, Amsterdam University Medical Centers, Location Academic Medical Center, University of Amsterdam, Amsterdam, The Netherlands; 9 Division of Infectious Diseases, Amsterdam University Medical Centers, Location Academic Medical Center, University of Amsterdam, Amsterdam, The Netherlands

**Keywords:** Pneumonia, Host immune response, Intensive care unit, Delayed admission

## Abstract

**Background:**

A delay in admission to the intensive care unit (ICU) of patients with community-acquired pneumonia (CAP) has been associated with an increased mortality. Decisions regarding interventions and eligibility for immune modulatory therapy are often made at the time of admission to the ICU. The primary aim of this study was to compare the host immune response measured upon ICU admission in CAP patients admitted immediately from the emergency department (direct ICU admission) with those who were transferred within 72 h after admission to the general ward (delayed ICU admission).

**Methods:**

Sixteen host response biomarkers providing insight in pathophysiological mechanisms implicated in sepsis and blood leukocyte transcriptomes were analysed in patients with CAP upon ICU admission in two tertiary hospitals in the Netherlands.

**Results:**

Of 530 ICU admissions with CAP, 387 (73.0%) were directly admitted and 143 (27.0%) had a delayed admission. Patients with a delayed ICU admission were more often immunocompromised (35.0 versus 21.2%, *P* = .002) and had more malignancies (23.1 versus 13.4%, *P* = .011). Shock was more present in patients who were admitted to the ICU directly (46.6 versus 33.6%, *P* = .010). Delayed ICU admission was not associated with an increased hospital mortality risk (hazard ratio 1.25, 95% CI 0.89–1.78, *P* = .20). The plasma levels of biomarkers (*n* = 297) reflecting systemic inflammation, endothelial cell activation and coagulation activation were largely similar between groups, with exception of C-reactive protein, soluble intercellular adhesion molecule-1 and angiopoietin-1, which were more aberrant in delayed admissions compared to direct ICU admissions. Blood leukocyte transcriptomes (*n* = 132) of patients with a delayed ICU admission showed blunted innate and adaptive immune response signalling when compared with direct ICU admissions, as well as decreased gene expression associated with tissue repair and extracellular matrix remodelling pathways.

**Conclusions:**

Blood leukocytes of CAP patients with delayed ICU admission show evidence of a more immune suppressive phenotype upon ICU admission when compared with blood leukocytes from patients directly transferred to the ICU.

*Trial registration*: Molecular Diagnosis and Risk Stratification of Sepsis (MARS) project, ClinicalTrials.gov identifier NCT01905033.

**Supplementary Information:**

The online version contains supplementary material available at 10.1186/s13613-021-00930-5.

## Background

Community-acquired pneumonia (CAP) is the most common infection requiring intensive care in developed countries [1, 2]. The number of patients requiring therapy on the intensive care unit (ICU) has increased over the last decades, mostly in elderly and patients with comorbidities [3, 4]. Among hospitalized CAP patients in the U.S., one in five is admitted to the ICU [5]. Hospital mortality among patients with this severe form of CAP remains high, ranging from 25% to more than 50% [6, 7].

Disease progression in severe CAP, from first symptoms to the need for supportive care such as mechanical ventilation or vasopressors, can vary widely between patients. This is reflected in the fact that some patients are transferred from the emergency department (ED) directly to the ICU, while up to 45% of those who eventually require ICU admission, is initially admitted to the general ward [8]. This so-called “delayed” ICU admission has been associated with an increased hospital length of stay and a higher mortality [9–11]. The term “delayed” implies that the severity of disease could have been recognized earlier; therefore, several scoring systems have been established, of which the IDSA/ATS 2007 criteria may be the strongest tool to predict ICU admission requirement [4].

Knowledge of the impact of delayed ICU admission on the pathophysiology of CAP is limited. Previous studies focused on differences between CAP patients with immediate or delayed ICU referral regarding premorbid conditions, reporting that those with a delayed ICU admission were older and more likely to have comorbidities such as cerebrovascular disease and diabetes mellitus [9, 10]. Another investigation compared the clinical presentation of CAP patients with an immediate or delayed ICU admission upon ED presentation, showing that patients who were transferred to the ICU directly had more aberrant clinical signs, including a lowered mental status, tachypnea, tachycardia and acidosis [10]. Thus far, studies comparing CAP patients with immediate or delayed ICU admission upon entrance in the ICU have not been reported. Furthermore, the immune response in patients with delayed ICU referral has not been investigated. Knowledge about the relationship between delayed ICU admission and distinct features of host response aberrations may lead to a better understanding of the immunopathology associated with severe CAP and may guide future identification of patients who are more likely to benefit from immunomodulatory therapies upon ICU admission.

We hypothesized that CAP patients with delayed ICU admission show a more blunted immune response relative to those with direct ICU admission due to a more protracted disease course. To test this hypothesis, we compared immune responses in CAP patients who were transferred immediately from the ED to the ICU (direct admission) to those who were first admitted to the ward before being admitted to the ICU within 3 days (delayed admission). To obtain insight into differences in immunopathology, we measured 16 biomarkers proving insight into key pathways implicated in the pathogenesis of severe CAP and analysed the genome-wide transcriptomes in blood leukocytes in an unbiased way.

## Methods

### Study design, setting and patient identification

This study was conducted as part of the “Molecular Diagnosis and Risk Stratification of Sepsis” (MARS) project, a prospective observational cohort study in the ICUs of two tertiary teaching hospitals (Academic Medical Center in Amsterdam and University Medical Center in Utrecht) in the Netherlands (ClinicalTrials.gov identifier NCT01905033) [12, 13]. Between January 2011 and January 2014, all patients above 18 years of age admitted with an expected length of longer than 24 h were included via an opt-out method approved by the medical ethical committees of the participating hospitals.

CAP was diagnosed by the clinical team and subsequently reassessed and categorized in a four-point scale (ascending from none, possible, probable to definite) by dedicated research nurses, as described before [12, 14]. For the current analysis, all consecutive patients with CAP with a likelihood of at least *possible* were included. Exclusion criteria were patients with a hospital admission of more than 72 h prior to ICU admission, surgical admissions, readmissions within the same hospital stay or within 30 days after discharge, and transfers from another ICU, medium care facility, coronary care facility or unknown admission sources.

Direct ICU admission was defined as immediate admission from the ED to the ICU, whereas delayed ICU admission was defined as initial admission to the general ward, before ICU transfer. Decision to transfer patients to the ICU was taken by the treating physician and not influenced by the current study.

### Clinical variables

Dedicated research physicians prospectively collected demographic data, comorbidities and daily physiological measurements. Cardiovascular insufficiency was defined as a medical history of congestive heart failure, chronic cardiovascular disease, peripheral vascular disease or cerebrovascular disease. Immunocompromised state was defined as a medical history of immune deficiency, human immune deficiency virus (HIV) infection or acquired immune deficiency syndrome (AIDS), or by the use of corticosteroids or antineoplastic medication. Malignancy was defined as a medical history of either non-metastatic solid tumour, metastatic malignancy or hematologic malignancy. Renal insufficiency was defined as a history of chronic renal insufficiency, or treatment with chronic intermittent haemodialysis or continuous ambulatory peritoneal dialysis. Respiratory insufficiency was defined as chronic obstructive pulmonary disease or respiratory insufficiency in the medical history. Chronic comorbid conditions were scored using the Charlson comorbidity index [15].

Daily severity scores such as Acute Physiology and Chronic Health Evaluation (APACHE) IV and Sequential Organ Failure Assessment (SOFA) scores were calculated. Shock was defined by the use of vasopressors (norepinephrine, epinephrine or dopamine) in a norepinephrine-equivalent dose of more than 0.1 µg/kg/min. Acute kidney injury and acute respiratory distress syndrome were defined using strict pre-set criteria [16, 17]. ICU-acquired complications were defined when they occurred more than 48 h after ICU admission.

### Plasma biomarker measurements

Biomarkers indicative of host response pathways implicated in sepsis pathogenesis were measured on admission to the ICU in the subset of CAP patients with a definite or probable infection likelihood enrolled during the first 2.5 years. EDTA anticoagulated blood was collected on admission and stored within 4 h at -80 °C until use. Interleukin (IL)-6, IL-8, IL-10, soluble E-selectin, soluble intercellular adhesion molecule-1 (sICAM-1) and fractalkine were measured by FlexSet cytometric bead array (BD Biosciences, San Jose, CA) using FACSCalibur flow cytometer (Becton Dickenson, Franklin Lakes, NJ). Matrix metalloproteinase-8 (MMP-8), angiopoietin-1, angiopoietin-2, protein C, antithrombin, (all R&D systems, Abingdon, UK) and D-dimer (Procartaplex, eBioscience, San Diego, CA) were measured by Luminex multiplex assay using BioPlex 200 (BioRad, Hercules, CA). C-reactive protein (CRP) was determined by immunoturbidimetric assay (Roche diagnostics). Prothrombin time (PT) and activated partial thromboplastin time (APPT) were determined by using a photometric method with Dade Innovin Reagent or by Dade Actin FS Activated PTT Reagent, respectively (both Siemens Healthcare Diagnostics). Normal plasma protein values were acquired from EDTA plasma from 27 age- and gender-matched healthy volunteers (from whom written informed consent was obtained), with the exception of CRP, PT and APTT for which routine laboratory reference values were used.

### Blood gene expression microarrays

Whole blood was drawn in PAXgene™ tubes (Becton–Dickinson, Breda, the Netherlands) within 24 h after ICU admission. PAXgene™ blood samples were also collected from 42 healthy controls [median age 35 years (interquartile range 30–63); 57% male] after obtaining written informed consent. Total RNA was extracted using the PAXgene blood mRNA kit (Qiagen, Venlo, the Netherlands), according to manufacturer's instructions. Total RNA (RNA integrity number > 6.0) was processed and hybridized to the Affymetrix Human Genome U219 96-array and scanned by using the GeneTitan instrument at the Cologne Center for Genomics (CCG, Cologne, Germany), as described by the manufacturer (Affymetrix).

Raw data scans (.CEL files) were read into the R language and environment for statistical computing (version 2.15.1; R Foundation for Statistical Computing, Vienna, Austria; http://www.R-project.org/). Pre-processing and quality control were performed by using the Affy package (version 1.36.1) [18]. Array data were background corrected by robust multi-array average, quantile-normalized and summarized by median polish using the expresso function. The resultant 49,386 log-transformed probe intensities were filtered by means of a 0.5 variance cutoff using the genefilter method [19] to recover 24,646 expressed probes in at least one sample. The occurrence of non-experimental chip-effects was evaluated by means of the Surrogate Variable Analysis (R package version 3.4.0) and corrected by the empirical Bayes Method ComBat [20, 21]. The non-normalized and normalized MARS gene expression data sets are available at the Gene Expression Omnibus public repository of NCBI under the accession number GSE65682.

The 24,646 probes were assessed for differential abundance across healthy subjects and patient samples using the limma method (version 3.36.5) [22]. Supervised analysis (comparison between pre-defined groups) was performed by moderated t-statistics. We adjusted for the potential effect of age and gender in our model (additive). Throughout Benjamini–Hochberg (BH) multiple comparison adjusted probabilities, correcting for the 24,646 probes (false discovery rate < 5%), defined significance. Ingenuity pathway analysis (Ingenuity Systems IPA, http://www.ingenuity.com) was used to identify the association with canonical signalling pathways, stratifying genes by over- and under-expressed patterns using fold changes. The ingenuity knowledgebase was selected as reference and human species specified. All other parameters were default.

### Statistical analysis

All categorical variables are presented as numbers (percentages), parametric continuous variables are presented as means ± standard deviation (SD) and nonparametric continuous variables are presented as median and interquartile ranges (IQR, 25th and 75th percentiles). Data distribution was assessed by the Shapiro–Wilk test. A Mann–Whitney *U* or a Kruskal–Wallis test was used to analyse continuous nonparametric data, whereas continuous parametric data were analysed using a Student’s *t*-test or analysis of variance (two-sided analysis of variance). All categorical data were analysed using a Chi-square or Fisher exact test. Cox proportional hazard analysis of hospital mortality was performed using the Survival R package (version 3.2–7). Aside from CRP, platelets, PT and APTT, all plasma protein levels were log10 transformed for plotting purposes; all biomarkers were analysed using a Mann–Whitney* U* test. All analyses were performed in R studio version 4.0.2 (R Core Team 2013, Vienna, Austria). A nominal *P* value < 0.05 was considered to be of statistical significance for clinical data. Comparison of plasma biomarker levels were adjusted for multiple testing within each pathophysiological domain with the BH false discovery rate approach.

## Results

### Patients characteristics

**Fig. 1:**
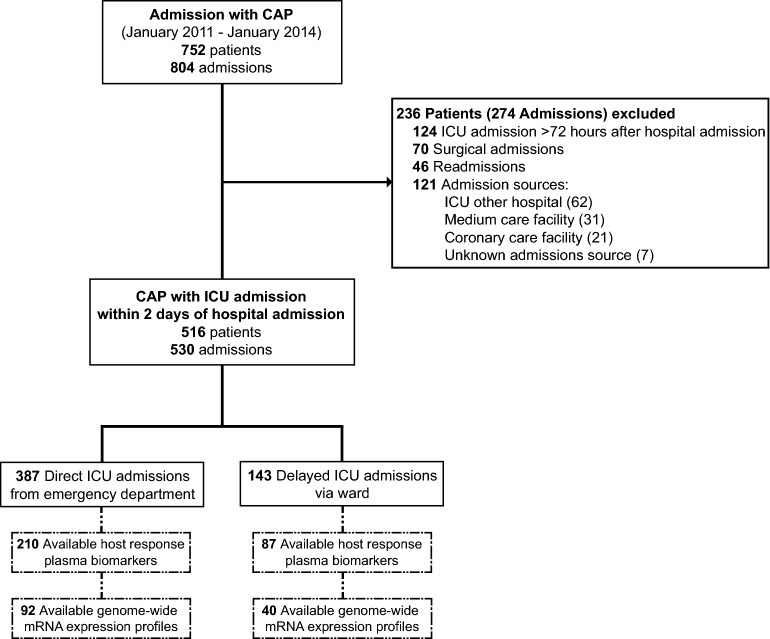
Flowchart of patient inclusion. In 75 admissions multiple exclusion criteria were met. *CAP* community-acquired pneumonia, *ICU* intensive care unit

A total of 804 ICU admissions with CAP were included in the 3-year study period (Fig. 1). Of these, 274 admissions (34.1%) were excluded because the ICU transferal took place more than 72 h after hospital admission, surgical admissions, readmissions and admissions not directly from the ED or via the ward (i.e. ICU other hospital, medium care facility, coronary care facility or unknown admission source). Of the remaining 530 admissions, 387 (73.0%) were directly admitted from the ED, and 143 (27.0%) had a delayed ICU admission via the ward.

Compared to patients with direct transferal to ICU, patients with delayed ICU admission differed minimally in terms of demographics and chronic comorbidities (Table 1). Patients with delayed ICU admission were more often immunocompromised (*P* = 0.002) and had more malignancies (*P* = 0.011). Nevertheless, the Charlson comorbidity index was not different between groups. The severity of disease upon ICU admission was also largely comparable in delayed and direct admissions, as reflected by similar APACHE IV and SOFA scores, as well as similar percentages of mechanical ventilation requirement. However, shock was more present in patients who were admitted directly (46.6 versus 33.6% after delayed admissions, *P* = 0.010). Causative pathogens, identified in slightly over half of all patients, did not differ between groups.

**Table 1:** Baseline characteristics and outcomes

	Direct ICU admission	Delayed ICU admission	*P* value
Patients	387	143	
Demographics			
Age, years, median [IQR]	62 [49, 72]	64 [51, 73]	.47
Gender male, n (%)	252 (65.1)	83 (58.0)	.26
White race, n (%)	344 (88.9)	123 (86.0)	.45
Readmission^a^, n (%)	11 (2.8)	3 (2.1)	.87
Chronic comorbidity, n (%)			
None	110 (28.4)	35 (24.5)	.43
Immunocompromised state	82 (21.2)	50 (35.0)	.002
Cardiovascular insufficiency	110 (28.4)	32 (22.4)	.20
Malignancy	52 (13.4)	33 (23.1)	.011
Renal insufficiency	41 (10.6)	19 (13.3)	.48
Respiratory insufficiency	114 (29.5)	35 (24.5)	.31
COPD	82 (21.2)	26 (18.2)	.52
Diabetes mellitus	86 (22.2)	24 (16.8)	.21
Charlson comorbidity index	4 [2, 6]	4 [3, 6]	.18
Vital signs on admission, median [IQR]			
Temperature	38 [37, 38]	38 [37, 39]	.14
PaO_2_/FiO_2_ ratio	114 [57, 477]	102 [50, 351]	.21
PEEP during first 24 h, cm H_2_O	24 [7, 25]	24 [6, 24]	.47
Severity of disease on ICU admission			
APACHE IV Score, median [IQR]	75 [59, 98]	75 [60, 92]	.75
SOFA total, median [IQR]	6 [4, 8]	6 [4, 8]	.69
Mechanical ventilation, n (%)	303 (78.3)	101 (70.6)	.08
Shock, n (%)	179 (46.6)	48 (33.6)	.010
Acute kidney injury, n (%)	121 (31.3)	36 (25.2)	.21
Acute respiratory distress syndrome, n (%)	95 (24.5)	36 (25.2)	.97
Acute myocardial infarction, n (%)	14 (3.6)	3 (2.1)	.55
Causative pathogens			.08
Gram-positive bacteria	68 (17.6)	17 (11.9)	
Gram-negative bacteria	57 (14.7)	24 (16.8)	
Atypical bacteria	1 (0.3)	1 (0.7)	
Virus	16 (4.1)	12 (8.4)	
Fungi	11 (2.8)	10 (7.0)	
Other pathogens	1 (0.3)	1 (0.7)	
Multiple pathogens	42 (10.9)	11 (7.7)	
Unknown	191 (49.4)	67 (46.9)	
Outcome			
Length of ICU stay, days, median [IQR]	5 [3, 9]	5 [2, 9]	.79
Length of hospital stay, days, median [IQR]	12 [16, 24]	14 [7, 24]	.21
MV characteristics			
Duration of initial MV, days, median [IQR]	2 [1, 6]	2 [1, 6]	.34
Recurrence of MV, n (%)	10 (2.6)	10 (7.0)	.035
MV-free days^b^, median [IQR]	22 [5, 26]	21 [5, 25]	.39
ICU-acquired complications, n (%)			
None	327 (84.5)	122 (85.3)	.92
Acute kidney injury	35 (9.0)	11 (7.7)	.75
Acute respiratory distress syndrome	22 (5.7)	7 (4.9)	.89
Infection	25 (6.5)	11 (7.7)	.76
Mortality^c^, n (%)			
ICU	71 (18.9)	27 (19.3)	> .99
Hospital	101 (26.9)	46 (32.9)	.22
30 days	107 (28.5)	44 (31.4)	.58
60 days	123 (32.7)	53 (37.9)	.32
90 days	129 (34.3)	61 (43.6)	.07
1 year	158 (42.0)	71 (50.7)	.10

*APACHE* Acute Physiology and Chronic Health Evaluation, *COPD* chronic obstructive pulmonary disease, *ED* emergency department, *FiO*_*2*_ fraction of inspired oxygen, *ICU* intensive care unit, *IQR* interquartile range, *MV* mechanical ventilation, *PaO*_*2*_ partial pressure of oxygen in arterial blood gas analysis, *PEEP* positive end-expiratory pressure, *SOFA* Sequential Organ Failure Assessment

^a^Readmissions > 30 days after hospital discharge

^b^Days alive and free from MV on day 28 of ICU stay

^c^Mortality was calculated using the first ICU-admission for each patient; readmissions were not included in this analysis

### Outcomes

Outcomes such as length of hospital stay, ICU-acquired complications and duration of mechanical ventilation did not differ between patients with direct and delayed ICU admission. Moreover, the number of days alive and free from ventilation on day 28 of ICU stay was equal, but patients with a delayed ICU admission had to be returned on mechanical ventilation more often (7.0 versus 2.6% after direct ICU admission, *P* = 0.035). The proportions of both short- and long-term mortality were similar between direct and delayed ICU admissions. In a Cox proportional hazard analysis delayed ICU admission was not associated with an increased crude risk of hospital mortality (hazard ratio 1.25, 95% CI 0.89–1.78, *P* = 0.20). To align our investigation with previously published studies that reported an increased mortality after delayed ICU admission in cohorts that excluded immunocompromised patients [9, 10], we performed a subgroup analysis excluding these patients. Also in this cohort, the risk of hospital mortality was not increased in patients with delayed ICU admission (hazard ratio 1.33, 95% CI 0.88–2.02, *P* = 0.18).

### Plasma host response biomarkers

**Fig. 2:**
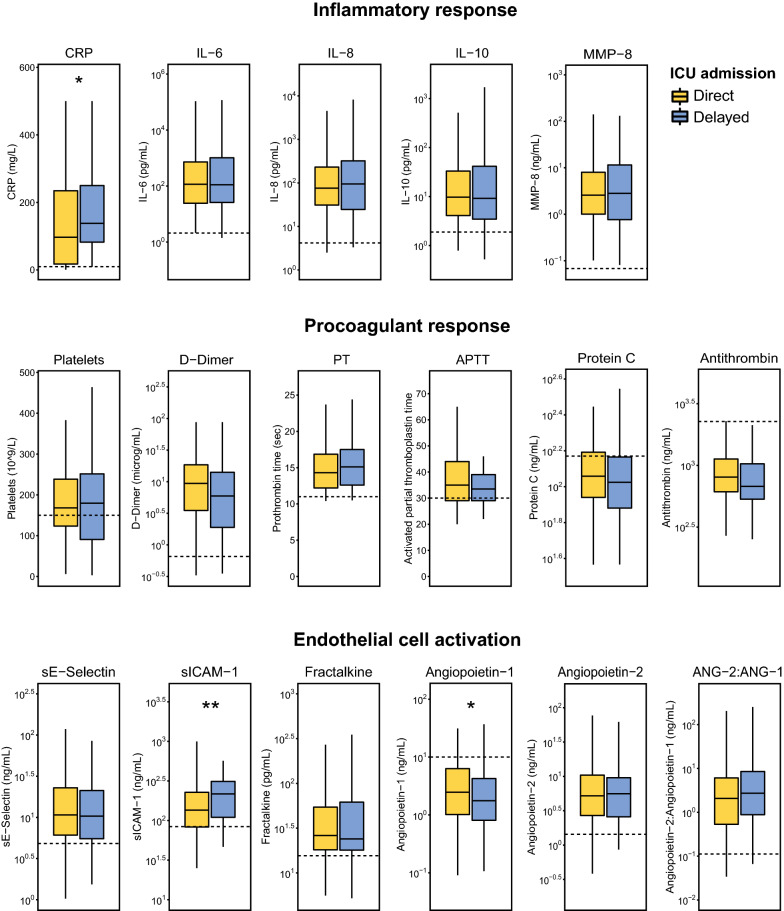
Host response plasma biomarkers in patients with community-acquired pneumonia with direct or delayed admission to the intensive care unit. Plasma biomarkers were measured on intensive care unit admission. Data are expressed as box-and-whisker plots depicting the median with the lower and upper quartiles. Upper and lower whiskers are defined as measurements that are within 1.5 times the interquartile range of the lowest and highest quartile. Dotted lines indicate median values obtained in 27 healthy subjects. *APTT* activated partial thromboplastin time, *CRP* C-reactive protein, *ICAM* intercellular adhesion molecule, *IL* interleukin, *MMP* matrix metalloproteinase, *PT* prothrombin time. Values in patients were all significantly different from those in healthy controls. * *P* < .05 (adjusted for multiple testing with the Benjamini–Hochberg false discovery rate approach)

Plasma biomarkers indicative of host response pathways involved in sepsis pathogenesis were measured on ICU admission in 210 direct and 87 delayed ICU admission patients (Fig. 1). This subgroup in which biomarkers were measured did not differ from the entire cohort with regard to baseline characteristics and outcomes (Additional file 1: Table S1). Compared to healthy controls, all patients admitted with CAP showed strong systemic inflammatory and cytokine responses (illustrated by higher concentrations of CCRP, IL-6, IL-8, IL-10 and MMP-8), activation of the coagulation system (increased D-Dimer, prolonged PT, prolonged APTT and reduced protein C and antithrombin levels) and endothelial cell activation and dysfunction (elevated soluble E-selectin, sICAM-1, fractalkine and angiopoietin-2/angiopoietin-1 ratio) (median values of healthy subjects shown as dotted lines in Fig. 2, all comparisons to the patient groups were significant (data not shown)). Plasma biomarker levels of patients with direct compared to those with delayed ICU admission were largely similar after multiple comparison adjustment, with exception of CRP (*P* = 0.018), and sICAM-1 (*P* = 0.005) and angiopoietin-1 (*P* = 0.043), which were more aberrant in patients with delayed ICU admissions compared to those with direct ICU admission. To account for the higher occurrence of an immunocompromised state in patients with a delayed ICU admission, we performed a sensitivity analysis excluding this group. Again, most plasma biomarkers were comparable between the cohorts, with higher levels of CRP (*P* = 0.0097) and sICAM-1 (*P* = 0.026) in patients with a delayed ICU admission (Additional file 2: Table S2). In this subgroup analysis, biomarkers of the procoagulant response were significantly more aberrant in delayed ICU admissions, with decreased plasma concentrations of protein C (*P* = 0.048) and antithrombin (*P* = 0.048).

### Blood leukocyte transcriptome analysis

**Fig. 3:**
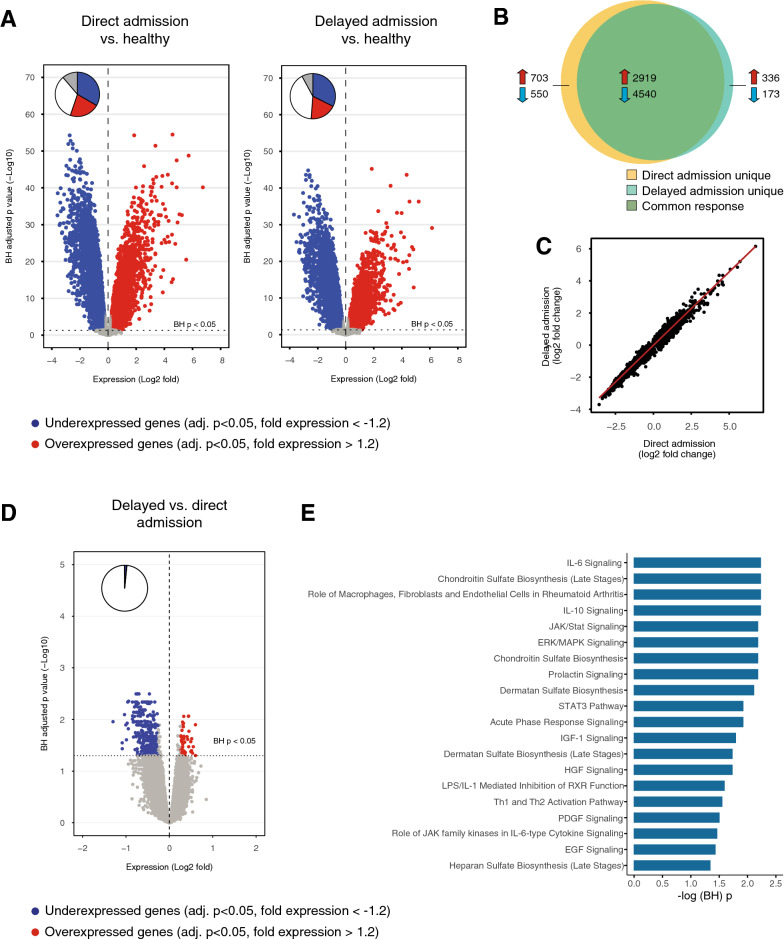
Leukocyte genomic responses in patients with community-acquired pneumonia with direct or delayed admission to the intensive care unit. **A** Volcano plots illustrating the differences in leukocyte genomic responses (integrating log2-fold changes and multiple-test adjusted probabilities) between patients with direct admission to the ICU for community-acquired pneumonia (CAP) and healthy subjects (left), and patients with delayed admission for CAP and healthy subjects (right). Considering adjusted *P* < .05, 8712 and 7968 genes were identified as differentially expressed in patients admitted directly of with delay for CAP vs healthy subjects, respectively. Blue dots represent significantly underexpressed genes (adjusted *P* < .05, fold expression < − 1.2), whereas red dots represent significantly overexpressed genes (adjusted* P* < .05, fold expression > 1.2) in patients relative to healthy controls. Horizontal dotted line indicates multiple-test adjusted Benjamini–Hochberg (BH) *P* < .05 threshold. Within plots, pie charts show the extent of gene expression changes: blue slices show significantly underexpressed genes (adjusted *P* < .05 and expression more than 1.2-times decreased compared with healthy controls), red slices show significantly overexpressed genes (adjusted *P* < .05 and expression more than 1.2-time increased compared with healthy controls), and grey slices show significantly different gene expression (adjusted *P* < .05 and expression less than 1.2-time increased or decreased compared with healthy controls). **B** Venn–-Euler representation of differentially expressed genes on admission in CAP patients with direct or delayed ICU-admission vs healthy subjects (adjusted *P* < .05). Red arrows denote overexpressed genes, blue arrows denote underexpressed genes. **C** Dot plot depicting the common response (log2-fold changes) of CAP patients with direct or delayed ICU-admission as compared with healthy subjects. Rho, Spearman’s correlation coefficient. **D** Volcano plot illustrating the differences in leukocyte genomic responses on admission between patients with delayed compared with direct admission to the ICU for community-acquired pneumonia (CAP). Considering adjusted *P* < .05, 268 genes were differentially expressed. **E** Considering Benjamini–Hochberg’s adjusted *P* < .05, underexpressed genes were analysed for association with canonical signalling pathways by Ingenuity pathway analysis (IPA, www.ingenuity.com). Pathways are stratified by significance, which was gauged by BH-adjusted Fisher exact probability. –log (BH) *P*, negative log transformed BH-adjusted *P* value. Within plots, pie charts show the extent of gene expression changes in delayed compared to direct admissions for pneumonia

In the subgroup of patients with CAP with the highest infection likelihood (definite or probable) enrolled during the first 1.5 years of the study period blood leukocyte genome-wide RNA profiles were determined on admission (n = 132, of whom 92 with direct ICU admission and 40 with delayed ICU admission) (Fig. 1, patient characteristics of this subcohort shown in Additional file 3: Table S3). Blood RNA profiles of patients were initially compared with those of 42 healthy controls. Both patient groups, with either direct of delayed ICU admission, displayed strong blood transcriptome alterations, encompassing 68–74% of all genes present on the array, relative to healthy controls (Fig. 3A). Of the altered transcriptomes, 81% were common between patients with direct or delayed ICU admission (Fig. 3B); this common response showed strongly correlated gene expression fold changes (Fig. 3C). Pathway analysis of the common response revealed overexpression of genes involved in both pro- (IL-1, IL-6, IL-8, TREM-1, Toll-like receptor and inflammasome signalling) and anti-inflammatory (IL-10, arginase pathway signalling) innate immune responses, and metabolic pathways (mitochondrial dysfunction, HIF-1α signalling). Concomitantly, genes involved in lymphocyte (B-cell development, Th1 and Th2 activation, T-cell receptor signalling pathways), antigen presentation and mTOR pathways were underexpressed (Additional file 5: Figure S1).

Differential gene expression analysis revealed differences between patient groups, encompassing 268 significantly altered genes in CAP patients with delayed ICU admission relative to those with direct ICU admission (multiple-comparison adjusted *P* < 0.05) (Fig. 3D). Pathway analysis showed that underexpressed genes in patients with delayed relative to direct ICU-admission were significantly associated to blunted innate (IL-6, IL-10, STAT3, ERK/MAPK, acute phase response signalling) and adaptive (Th1 and Th2 activation pathway) immune response signalling, tissue repair and extracellular matrix remodelling (insulin-like growth factor-1, hepatocyte-, platelet-derived-, and epidermal growth factor signalling, as well as chondroitin sulfate, dermatan sulfate, heparan sulfate biosynthesis) (Fig. 3E). Overexpressed genes were not significantly associated to any specific pathway.

In the subgroup in which immunocompromised patients were excluded (comparing 68 direct admissions and 23 delayed admissions), the transcriptome profiles remained strongly altered as compared to healthy controls (Additional file 6: Figure S2A) and showed a largely overlapping response between patient groups with 80% commonality between the altered transcriptomes (Additional file 6: Figure S2B) and a strong correlation in gene expression (Additional file 6: Figure S2C). The direct comparison of delayed and direct ICU admission showed a lower number of 111 significantly altered genes (Additional file 6: Figure S2D), which were not significantly associated with specific pathways, probably due to the lower sample size used for the analysis. In support of this notion the main significantly differentially expressed genes associated with the downregulated immunological pathways in all delayed ICU admissions (Fig. 3E) were still underexpressed in the delayed ICU admission cohort without immunocompromised patients (e.g. SOCS3, IL4R, MAP2K1, REL, SHC1; Additional file 4: Table S4).

## Discussion

Patients with CAP who during their disease course require intensive care, are transferred to the ICU either directly from the ED or with a delay when treatment on a general ward falls short. Whilst several studies reported differences between patients with direct or delayed ICU admission upon their first presentation to the ED, little is known about possible disparities between these groups upon entrance into the ICU. The primary aim of this study was to compare the immune response upon ICU admission in CAP patients with a delayed ICU admission to those who were transferred directly from the ED. We considered this of interest since a more protracted course of severe infection and sepsis may affect host response aberrations [2, 23, 24] and decisions about interventions that seek to modulate the host response or the eligibility of patients for immune modulatory trials are oftentimes made shortly after ICU admission [25, 26]. We show that, whilst overall the host response was largely similar between these two patient groups, analysis of genome-wide mRNA expression profiles established that blood leukocytes of patients with a delayed ICU admission display blunted innate and adaptive immune response signalling, as well as decreased expression of genes associated with tissue repair and extracellular matrix remodelling pathways.

Various factors can lead to delayed ICU admission after ED presentation for CAP, including organizational issues such as lack of ICU capacity [27] and overcrowding in the ED [28]. Several studies investigating the impact of deferred ICU admission defined “delayed” as 6–24 h after ED presentation [29–31]. In our study, we sought to investigate delays in ICU admission that were more likely related to less severe disease on ED presentation with later deterioration, rather than by organizational factors. Therefore, we defined delayed ICU admissions as those involving patients that were first admitted to the ward and subsequently transferred to the ICU within a relative wide time window of 72 h; we chose not to study patients admitted to the ICU after more than 72 h in order to maintain a reasonable connection between the primary reason for ED presentation and the subsequent need for intensive care. This is in line with previous studies which used the same time window of 72 h [9, 10]. Using these boundaries we found that 27% of CAP patients admitted to the ICU were first admitted to a general ward, which is less than the 48% in the study of Phua et al. [9], but comparable to the 30% in the cohort of Renaud et al. [10].

Patients with a delayed ICU admission were more likely to be immunocompromised or have a history of malignancy. Previous studies did not report this difference; however, two investigations excluded immunocompromised patients [9, 10], and the incidence of neoplastic disease was relatively low [9–11]. In our cohort, the overall incidence of cancer was 16% (23.1% in the delayed admission group versus 13.4% in direct admissions), while in the other studies the proportion of cancer patients was not higher than 10.6%. This difference could be explained by the fact that our study was conducted in two tertiary hospitals, where many cancer patients are treated. Our investigation does not provide insight into the reasons why immune compromise and cancer are associated with delayed ICU admission in patients with CAP, although in case of cancer, physicians may be less keen to transfer patients to the ICU. However, subgroup analyses excluding immunocompromised patients revealed that the main differences in the host immunological response between patients with a delayed and direct ICU admission are independent of an immunocompromised state.

This study is the first to compare the clinical characteristics of CAP patients with direct and delayed ICU admission upon entrance into the ICU. Previous studies focussed on clinical features upon presentation in the ED, showing that CAP patients with a direct ICU admission were more severely ill, as illustrated by a higher proportion of patients with an altered mental state, tachypnea, hypotension and acidosis [9, 10]. We here show that on ICU admission patients with immediate or delayed ICU referral had similar severities of disease as indicated by APACHE IV and SOFA scores, and the frequency of AKI and ARDS. Of note, patients with delayed ICU admission less often had shock, which could be explained by medical care on the general ward prior to ICU admission.

Previous studies on delay of ICU admission reported an increased mortality in the overall ED population [29–31], as well as specifically in CAP patients [9–11]. In our cohort crude mortalities did not differ between CAP patients with direct or delayed ICU admission (hazard ratio 1.25, 95% CI 0.89–1.78, *P* = 0.20). Other studies found greater differences in mortality between CAP patients with direct or delayed ICU admission [9, 11]; however, herein mortality in the delayed admission group was much higher compared to our cohort (i.e. hospital mortality of 51% [9] and 30-day mortality of 47% [11], compared to 26.9 and 31.4%, respectively, in our cohort). While earlier investigations excluded immunocompromised patients [9, 10], we here demonstrate that this exclusion did not significantly modify the risk for hospital mortality in patients with delayed relative to direct ICU admission (hazard ratio 1.33, 95% CI 0.88–2.02, *P* = 0.18).

By measuring 16 plasma biomarkers providing insight into key pathophysiologic mechanisms in sepsis, we show that on ICU admission the host immune response between CAP patients with delayed or direct ICU referral is largely similar. However, the plasma levels of CRP and sICAM-1 were increased in the delayed ICU admission group, and angiopoietin-1 was decreased. Méndez et al. reported that CAP patients with a duration of symptoms longer than 3 days had higher CRP, but lower IL-6 and IL-8 levels upon admission to the general ward than those with a shorter duration of symptoms [32]. Thus, the higher CRP levels in patients with delayed ICU admission might be related to a longer duration of disease, although our study is limited by the lack of information of the onset of disease prior to presentation in the ED. Of note, however, all other biomarkers of inflammation and coagulation were similar between groups, suggesting that these host response aberrations, which frequently have been targeted in immune modulatory trials in critically ill patients with CAP and/or sepsis [25, 26, 33], are not influenced by a 1- to 3-day delay of ICU admission. Likewise, biomarkers of endothelial cell activation and dysfunction were not different between patients with immediate or delayed ICU admission, with the exception of sICAM-1 and angiopoietin-1, which reflect activation and stabilization of the vascular endothelium, respectively. Although both circulating sICAM-1 and angiopoietin-1 levels have been shown to be correlated with disease severity and increased mortality in CAP [34] and sepsis [35, 36], in our study disease severity or mortality did not differ between patients with direct and delayed ICU admission. The functional implications of higher circulating sICAM-1 in patients with delayed ICU admission remain to be established and are likely (at least in part) dependent on tissue levels. sICAM-1 also reflects leukocyte activation, and at high levels can inhibit interactions between leukocytes and endothelial cells and promote repair activity of immune cells, whilst at low concentrations sICAM-1 can enhance inflammation through activation of NF*κ*B [37].

Unbiased investigation of the immune response by determining gene expression profiles of blood leukocytes revealed that delayed ICU admission was associated with blunted adaptive and innate immune response signalling on ICU admission, which is suggestive of a more immune suppressive phenotype relative to patients with direct ICU admission, possibly related to a more prolonged disease course [24, 38]. This finding may indicate that CAP patients with a delayed ICU admission are more likely to benefit from immune stimulatory treatment [24, 38]. Predictive enrichment of study populations through selection of patients who are more likely to respond favourably to a certain therapy has been advocated as an important tool for future immune modulatory sepsis trials [24, 39]. Our investigation suggests that delayed ICU admission, apart from sophisticated immunological measurements, could be a clinical parameter for predictive enrichment of immune stimulatory trials in severe CAP and/or sepsis. Genes encoding proteins implicated in tissue repair and extracellular matrix remodelling were also underexpressed in patients with delayed ICU admission relative to those with direct ICU admission. In this context, we found that leukocytes from patients with a delayed ICU admission had a reduced expression of genes involved in chondroitin sulphate and dermatan sulphate biosynthesis, glycosaminoglycans with important roles in tissue repair in the lung [40]. Moreover, pathways involving growth factors have also been associated with lung tissue remodelling [41], of which we found insulin-like growth factor-1 [42], hepatocyte- [43], platelet-derived- [44], and epidermal growth factor signalling [45] to be downregulated in leukocytes of delayed ICU admission patients.

Our study has strengths and limitations. This is the first study to determine and compare clinical characteristics and the host immune response in CAP patients with a delayed and direct ICU admission upon entrance to the ICU. We measured a large set of host response plasma biomarkers and performed whole-genome blood leukocyte transcriptome analysis in a large prospectively enrolled study population. Our study was done in two tertiary hospitals in the Netherlands and may not be representative for other settings and/or areas in the world. We did not capture information about the duration of symptom prior to ED presentation.

## Conclusions

CAP patients with a delayed ICU referral via a general ward are admitted to the ICU with largely similar severity of disease and plasma host response biomarker profiles as compared to patients admitted directly from the ER. However, blood leukocytes of patients with delayed ICU admission demonstrate evidence of blunted innate and adaptive immune response signalling, suggestive of a more immune suppressive phenotype.

## Supplementary Information

**Additional file 1: Table S1**. Baseline characteristics and outcome of patients with community-acquired pneumonia with direct or delayed admission to the intensive care unit, included in the analysis of host response plasma biomarkers.

**Additional file 2: Table S2**. Host response plasma biomarkers in patients with community-acquired pneumonia with direct or delayed admission to the intensive care unit, immunocompromised patients excluded.

**Additional file 3: Table S3**. Baseline characteristics and outcome of patients with community-acquired pneumonia with direct or delayed admission to the intensive care unit, included in the blood whole genome analyses.

**Additional file 4: Table S4**. List of significantly altered genes in patients with a delayed ICU admission, as compared to those with a direct ICU admission, all immunocompromised patients excluded.

**Additional file 5: Figure S1**. Common transcriptional response in blood leukocytes obtained on admission in patients with direct or delayed ICU-admission for community-acquired pneumonia relative to health.

**Additional file 6: Figure S2**. Leukocyte genomic responses in patients with community-acquired pneumonia with direct or delayed admission to the intensive care unit, all immunocompromised patients excluded.

## Data Availability

The datasets analysed during the current study are available from the corresponding author on reasonable request.

## Abbreviations

AIDS: Acquired immunodeficiency syndrome
APACHE-IV: Acute Physiology and Chronic Health Evaluation IV
aPTT: Activated partial thromboplastin time
BH: Benjamini–Hochberg
BMI: Body mass index
CAP: Community-acquired pneumonia
COPD: Chronic obstructive pulmonary disease
CCG: Cologne Center for Genomics
CRP: C-reactive protein
ED: Emergency department
EDTA: Ethylenediaminetetraacetic acid
HIV: Human immunodeficiency virus
HR: Hazard ratio
ICU: Intensive care unit
IDSA/ATS: Infectious Diseases Society of America/American Thoracic Society
IL: Interleukin
IPA: Ingenuity Pathway Analysis
IQR: Interquartile ranges
MARS: Molecular Diagnosis and Risk Stratification of Sepsis
MMP-8: Matrix metalloproteinase-8
MV: Mechanical ventilation
PEEP: Positive end-expiratory pressure
PT: Prothrombin time
RNA: Ribonucleic acid
SD: Standard deviation
sICAM-1: Soluble intercellular adhesion molecule-1
SOFA: Sequential Organ Failure Assessment
