# Supplementary material for: Association between delay in intensive care unit admission and the host response in patients with community-acquired pneumonia
Source: Ann Intensive Care. 2021 Sep 28;11:142. doi: 10.1186/s13613-021-00930-5 (PMC8478267; doi:10.1186/s13613-021-00930-5)
Supplement: Supplementary file 1 — Additional file 1: Table S1. Baseline characteristics and outcome of patients with community-acquired pneumonia with direct or delayed admission to the intensive care unit, included in the analysis of host response plasma biomarkers. [file 13613_2021_930_MOESM1_ESM.docx]

***Table E1. Baseline characteristics and outcome of patients with community-acquired pneumonia with direct or delayed admission to the intensive care unit, included in the analysis of host response plasma biomarkers***

|  | **Direct**  **ICU admission** | **Delayed**  **ICU admission** | **P value** |
| --- | --- | --- | --- |
| **Patients** | **210** | **87** |  |
| **Demographics** |  |  |  |
| Age, years, median [IQR] | 63 [51, 72] | 64 [52, 72] | .97 |
| Gender male, n (%) | 142 (67.6) | 50 (57.5) | .13 |
| White race, n (%) | 184 (87.6) | 75 (86.2) | .89 |
| Readmission^a^, n (%) | 8 (3.8) | 2 (2.3) | .76 |
| Chronic comorbidity, n (%) |  |  |  |
| None | 51 (24.3) | 21 (24.1) | >.99 |
| Immunocompromised state | 52 (24.8) | 36 (41.4) | .007 |
| Cardiovascular insufficiency | 67 (31.9) | 19 (21.8) | .11 |
| Malignancy | 27 (12.9) | 21 (24.1) | .026 |
| Renal insufficiency | 24 (11.4) | 15 (17.2) | .25 |
| Respiratory insufficiency | 63 (30.0) | 25 (28.7) | .94 |
| COPD | 49 (23.3) | 18 (20.7) | .73 |
| Diabetes Mellitus | 53 (25.2) | 15 (17.2) | .18 |
| Charlson comorbidity index | 4 [2, 6] | 4 [3, 6] | .71 |
| Vital signs on admission, median [IQR] |  |  |  |
| Temperature | 38 [37, 38] | 38 [37, 39] | .21 |
| PaO2/FiO2 ratio | 105 [47, 483] | 98 [30, 257] | .32 |
| PEEP during first 24 hours, cm H2O | 14 [6, 24] | 10 [5, 24] | .78 |
| Severity of disease on ICU admission |  |  |  |
| APACHE IV Score, median [IQR] | 77 [60, 105] | 79 [60, 92] | .79 |
| SOFA Total, median [IQR] | 7 [5, 9] | 6 [4, 9] | .23 |
| Mechanical ventilation, n (%) | 179 (85.2) | 70 (80.5) | .40 |
| Shock, n (%) | 110 (52.4) | 34 (39.1) | .05 |
| Acute kidney injury, n (%) | 77 (36.7) | 28 (32.2) | .55 |
| Acute respiratory distress syndrome, n (%) | 75 (35.7) | 29 (33.3) | .80 |
| Acute myocardial infarction, n (%) | 8 (3.8) | 1 (1.1) | .40 |
| **Causative pathogen** |  |  | .14 |
| Gram-positive bacteria | 41 (19.5) | 14 (16.1) |  |
| Gram-negative bacteria | 32 (15.2) | 18 (20.7) |  |
| Atypical bacteria | 1 (0.5) | 1 (1.1) |  |
| Virus | 10 (4.8) | 9 (10.3) |  |
| Fungi | 10 (4.8) | 7 (8.0) |  |
| Other pathogens | 0 (0.0) | 1 (1.1) |  |
| Multiple pathogens | 29 (13.8) | 7 (8.0) |  |
| Unknown | 87 (41.4) | 30 (34.5) |  |
| **Outcome** |  |  |  |
| Length of ICU stay, days, median [IQR] | 5 [3, 11] | 6 [3, 10] | .83 |
| Length of hospital stay, days, median [IQR] | 14 [8, 30] | 14 [8, 26] | .90 |
| MV characteristics |  |  |  |
| Duration of initial MV, days, median [IQR] | 3 [1, 7] | 3 [1, 7] | .56 |
| Recurrence of MV, n (%) | 7 (3.3) | 9 (10.3) | .031 |
| MV-free days^b^, median [IQR] | 21 [4, 26] | 19 [3, 25] | .40 |
| ICU-acquired complications, n (%) |  |  |  |
| None | 163 (77.6) | 69 (79.3) | .87 |
| Acute kidney injury | 25 (11.9) | 9 (10.3) | .85 |
| Acute respiratory distress syndrome | 18 (8.6) | 5 (5.7) | .56 |
| Infection | 17 (8.1) | 10 (11.5) | .48 |
| **Mortality^c^, n (%)** |  |  |  |
| ICU | 39 (19.3) | 18 (21.2) | .84 |
| Hospital | 52 (25.7) | 28 (32.9) | .27 |
| 30 days | 56 (27.7) | 26 (30.6) | .73 |
| 60 days | 67 (33.2) | 31 (36.5) | .69 |
| 90 days | 71 (35.1) | 38 (44.7) | .16 |
| 1 year | 90 (44.6) | 45 (52.9) | .24 |
| Abbreviations: APACHE, Acute Physiology and Chronic Health Evaluation; COPD, chronic obstructive pulmonary disease; FiO_2_, fraction of inspired oxygen; ICU, intensive care unit; IQR, interquartile range; MV, mechanical ventilation; PaO_2,_ partial pressure of oxygen in arterial blood gas analysis; PEEP, positive end-expiratory pressure; SOFA, Sequential Organ Failure Assessment.  ^a^ Readmissions > 30 days after hospital discharge.  ^b^ Days alive and free from MV on day 28 of ICU stay.  ^c^ Mortality was calculated using the first ICU-admission for each patient; readmissions were not included in this analysis. | | | |
