# Supplementary material for: Association between delay in intensive care unit admission and the host response in patients with community-acquired pneumonia
Source: Ann Intensive Care. 2021 Sep 28;11:142. doi: 10.1186/s13613-021-00930-5 (PMC8478267; doi:10.1186/s13613-021-00930-5)
Supplement: Supplementary file 2 — Additional file 2: Table S2. Host response plasma biomarkers in patients with community-acquired pneumonia with direct or delayed admission to the intensive care unit, immunocompromised patients excluded. [file 13613_2021_930_MOESM2_ESM.docx]

***Table E2. Host response plasma biomarkers in patients with community-acquired pneumonia with direct or delayed admission to the intensive care unit, immunocompromised patients excluded.***

|  | **Direct**  **ICU admission** | **Delayed**  **ICU admission** | ***P* value** |
| --- | --- | --- | --- |
| Patients | 158 | 51 |  |
| **Inflammatory response** | | | |
| CRP (mg/ml) | 50.00 [5.75, 199.75] | 133.50 [79.00, 244.25] | .0097 |
| IL-6 (pg/ml) | 112.63 [23.37, 512.95] | 188.78 [42.84, 1620.64] | .36 |
| IL-8 (pg/ml) | 68.51 [31.43, 182.00] | 97.22 [31.30, 419.83] | .36 |
| IL-10 (pg/ml) | 10.29 [4.12, 32.75] | 8.29 [2.72, 43.12] | .82 |
| MMP-8 (ng/ml) | 2.33 [0.97, 7.83] | 3.64 [0.67, 14.69] | .36 |
| **Procoagulant response** | | | |
| Platelets | 176.00 [132.00, 240.50] | 184.00 [119.00, 253.00] | .87 |
| D-Dimer (µg/ml) | 9.02 [3.18, 18.62] | 8.28 [2.30, 15.48] | .64 |
| PT (sec) | 14.50 [12.20, 18.20] | 15.30 [12.75, 20.50] | .37 |
| APTT (sec) | 36.00 [29.00, 52.00] | 34.00 [29.00, 43.00] | .81 |
| Protein C (ng/ml) | 113.79 [83.37, 163.55] | 95.96 [73.12, 123.09] | .048 |
| Antitrombin (ng/ml) | 777.62 [593.51, 1050.25] | 629.22 [496.93, 944.84] | .048 |
| **Endothelial cell activation** | | | |
| sE-Selectin (ng/ml) | 10.67 [5.96, 23.36] | 12.69 [7.91, 24.27] | .42 |
| sICAM-1 (ng/ml) | 129.65 [82.47, 187.40] | 203.47 [108.21, 294.23] | .026 |
| Fractalkine (pg/ml) | 24.52 [15.92, 49.38] | 23.56 [17.45, 49.58] | .62 |
| ANG-1 (ng/ml) | 2.91 [1.26, 7.25] | 2.25 [0.97, 4.93] | .23 |
| ANG-2 (ng/ml) | 4.66 [2.28, 10.02] | 6.68 [3.77, 9.57] | .29 |
| ANG-2:ANG-1 ratio | 1.87 [0.44, 4.24] | 3.00 [0.80, 7.73] | .10 |

Plasma biomarkers were measured on intensive care unit admission. Data are expressed as median with the interquartile range. P values were adjusted for multiple testing with the Benjamini-Hochberg false discovery rate approach. Abbreviations: APTT, activated partial thromboplastin time; CRP, C-reactive protein; ICAM, intercellular adhesion molecule; IL, interleukin; MMP, matrix metalloproteinase; PT, prothrombin time.
