# Supplementary material for: Association between delay in intensive care unit admission and the host response in patients with community-acquired pneumonia
Source: Ann Intensive Care. 2021 Sep 28;11:142. doi: 10.1186/s13613-021-00930-5 (PMC8478267; doi:10.1186/s13613-021-00930-5)
Supplement: Supplementary file 3 — Additional file 3: Table S3. Baseline characteristics and outcome of patients with community-acquired pneumonia with direct or delayed admission to the intensive care unit, included in the blood whole genome analyses. [file 13613_2021_930_MOESM3_ESM.docx]

***Table E3. Baseline characteristics and outcome of patients with community-acquired pneumonia with direct or delayed admission to the intensive care unit, included in the blood whole genome analyses***

|  | **Direct**  **ICU admission** | **Delayed**  **ICU admission** | **P value** |
| --- | --- | --- | --- |
| **Patients** | **92** | **40** |  |
| **Demographics** |  |  |  |
| Age, years, median [IQR] | 64 [53, 72] | 64 [48, 73] | .82 |
| Gender male, n (%) | 62 (67.4) | 22 (55.0) | .24 |
| White race, n (%) | 78 (84.8) | 33 (82.5) | .94 |
| Readmission^a^, n (%) | 2 (2.2) | 0 (0.0) | .87 |
| Chronic comorbidity, n (%) |  |  |  |
| None | 23 (25.0) | 8 (20.0) | .69 |
| Immunocompromised state | 24 (26.1) | 17 (42.5) | .10 |
| Cardiovascular insufficiency | 27 (29.3) | 8 (20.0) | .37 |
| Malignancy | 17 (18.5) | 8 (20.0) | >.99 |
| Renal insufficiency | 11 (12.0) | 8 (20.0) | .35 |
| Respiratory insufficiency | 25 (27.2) | 12 (30.0) | .90 |
| COPD | 22 (23.9) | 10 (25.0) | >.99 |
| Diabetes Mellitus | 24 (26.1) | 8 (20.0) | .60 |
| Charlson comorbidity index | 4 [2, 6] | 4 [3, 6] | .61 |
| Vital signs on admission, median [IQR] |  |  |  |
| Temperature | 38 [37, 39] | 38 [37, 39] | .93 |
| PaO2/FiO2 ratio | 119 [56, 512] | 61 [27, 511] | .15 |
| PEEP during first 24 hours, cm H2O | 10 [5, 24] | 16 [5, 24] | .58 |
| Severity of disease on ICU admission |  |  |  |
| APACHE IV Score, median [IQR] | 82 [64, 108] | 80 [64, 92] | .38 |
| SOFA Total, median [IQR] | 8 [5, 9] | 6 [4, 10] | .07 |
| Mechanical ventilation, n (%) | 81 (88.0) | 32 (80.0) | .35 |
| Shock, n (%) | 51 (55.4) | 17 (42.5) | .24 |
| Acute kidney injury, n (%) | 32 (34.8) | 13 (32.5) | .96 |
| Acute respiratory distress syndrome, n (%) | 37 (40.2) | 12 (30.0) | .36 |
| Acute myocardial infarction, n (%) | 4 (4.3) | 0 (0.0) | .43 |
| **Causative pathogen** |  |  | .18 |
| Gram-positive bacteria | 23 (25.0) | 5 (12.5) |  |
| Gram-negative bacteria | 19 (20.7) | 9 (22.5) |  |
| Atypical bacteria | 1 (1.1) | 0 (0.0) |  |
| Virus | 3 (3.3) | 1 (2.5) |  |
| Fungi | 6 (6.5) | 4 (10.0) |  |
| Multiple pathogens | 17 (18.5) | 3 (7.5) |  |
| Unknown | 23 (25.0) | 18 (45.0) |  |
| **Outcome** |  |  |  |
| Length of ICU stay, days, median [IQR] | 8 [4, 12] | 6 [3, 10] | .60 |
| Length of hospital stay, days, median [IQR] | 15 [9, 30] | 15 [9, 24] | .83 |
| MV characteristics |  |  |  |
| Duration of initial MV, days, median [IQR] | 4 [1, 9] | 4 [2, 7] | .67 |
| Recurrence of MV, n (%) | 5 (5.4) | 3 (7.5) | .95 |
| MV-free days^b^, median [IQR] | 18 [4, 25] | 18 [5, 24] | .93 |
| ICU-acquired complications, n (%) |  |  |  |
| None | 67 (72.8) | 32 (80.0) | .51 |
| Acute kidney injury | 15 (16.3) | 4 (10.0) | .50 |
| Acute respiratory distress syndrome | 6 (6.5) | 3 (7.5) | >.99 |
| Infection | 10 (10.9) | 5 (12.5) | >.99 |
| **Mortality^c^, n (%)** |  |  |  |
| ICU | 17 (18.9) | 5 (12.5) | .52 |
| Hospital | 23 (25.6) | 12 (30.0) | .75 |
| 30 days | 25 (27.8) | 12 (30.0) | .96 |
| 60 days | 28 (31.1) | 15 (37.5) | .61 |
| 90 days | 31 (34.4) | 17 (42.5) | .50 |
| 1 year | 41 (45.6) | 21 (52.5) | .59 |
| Abbreviations: APACHE, Acute Physiology and Chronic Health Evaluation; COPD, chronic obstructive pulmonary disease; FiO_2_, fraction of inspired oxygen; ICU, intensive care unit; IQR, interquartile range; MV, mechanical ventilation; PaO_2,_ partial pressure of oxygen in arterial blood gas analysis; PEEP, positive end-expiratory pressure; SOFA, Sequential Organ Failure Assessment.  ^a^ Readmissions > 30 days after hospital discharge.  ^b^ Days alive and free from MV on day 28 of ICU stay.  ^c^ Mortality was calculated using the first ICU-admission for each patient; readmissions were not included in this analysis. | | | |
