# Supplementary material for: Association between delay in intensive care unit admission and the host response in patients with community-acquired pneumonia
Source: Ann Intensive Care. 2021 Sep 28;11:142. doi: 10.1186/s13613-021-00930-5 (PMC8478267; doi:10.1186/s13613-021-00930-5)
Supplement: Supplementary file 4 — Additional file 4: Table S4. List of significantly altered genes in patients with a delayed ICU admission, as compared to those with a direct ICU admission, all immunocompromised patients excluded. [file 13613_2021_930_MOESM4_ESM.docx]

***Table E4. List of significantly altered genes in patients with a delayed ICU admission, as compared to those with a direct ICU admission, in the subgroup excluding patients with immune suppression***

| **Gene Symbol** | **Log2 FC** | **adj.P.Val** |
| --- | --- | --- |
| *TINF2* | -0.7187 | 0.0008 |
| *BCL3* | -0.6688 | 0.0025 |
| *PFKFB3* | -0.7704 | 0.0043 |
| *ARID5A* | -0.6950 | 0.0085 |
| *SOCS3* | -1.2371 | 0.0094 |
| *HLX* | -0.7889 | 0.0094 |
| *TRIB1* | -0.5042 | 0.0105 |
| *ACVR1B* | -0.5513 | 0.0109 |
| *RABGEF1* | -0.7858 | 0.0109 |
| *TMEM131* | -0.6213 | 0.0127 |
| *CTDP1* | -0.6053 | 0.0162 |
| *IL4R* | -0.6601 | 0.0162 |
| *FLJ36031* | -0.8247 | 0.0164 |
| *ACSL3* | -0.7828 | 0.0164 |
| *JMJD6* | -0.7476 | 0.0164 |
| *RASA2* | -0.7553 | 0.0167 |
| *GPR132* | -0.4516 | 0.0167 |
| *FCAR* | -0.9020 | 0.0167 |
| *LGALS8* | -0.6805 | 0.0167 |
| *ANKHD1* | -0.3801 | 0.0167 |
| *HIF1A* | -0.8178 | 0.0167 |
| *OSM* | -0.7486 | 0.0167 |
| *FOSL2* | -0.7216 | 0.0167 |
| *CHSY1* | -0.8718 | 0.0177 |
| *HIPK2* | -0.8073 | 0.0181 |
| *SYTL3* | -0.8509 | 0.0191 |
| *PHF13* | -0.6428 | 0.0191 |
| *SERTAD3* | -0.3730 | 0.0192 |
| *AGFG1* | -0.6687 | 0.0192 |
| *CRISP3* | -1.5209 | 0.0197 |
| *PGS1* | -0.4945 | 0.0198 |
| *BRI3BP* | 0.3611 | 0.0204 |
| *RAB43* | -0.6272 | 0.0204 |
| *ZFPL1* | -0.2863 | 0.0204 |
| *CSRNP1* | -0.6732 | 0.0204 |
| *VAPA* | -0.8059 | 0.0204 |
| **Gene Symbol** | **Log2 FC** | **adj.P.Val** |
| *TFG* | -0.4354 | 0.0242 |
| *PHTF1* | -0.7207 | 0.0242 |
| *DVL3* | -0.4781 | 0.0242 |
| *CEBPD* | -0.4896 | 0.0242 |
| *KIF1B* | -0.5969 | 0.0242 |
| *CSNK1D* | -0.5179 | 0.0242 |
| *CLASP1* | -0.4220 | 0.0245 |
| *PRDM1* | -0.4094 | 0.0246 |
| *ZFAND5* | 0.5678 | 0.0260 |
| *C20orf43* | -0.3093 | 0.0260 |
| *LPCAT3* | -0.5154 | 0.0260 |
| *ZNF276* | -0.4333 | 0.0268 |
| *FGD4* | -0.4268 | 0.0268 |
| *C15orf37* | -0.6130 | 0.0268 |
| *JUNB* | -0.4506 | 0.0283 |
| *RLIM* | -0.4893 | 0.0288 |
| *BTG2* | -0.5286 | 0.0300 |
| *ATAD3C* | 0.2464 | 0.0304 |
| *SHC1* | -0.3058 | 0.0312 |
| *MTHFS* | -0.5168 | 0.0312 |
| *MAP2K1* | -0.5138 | 0.0321 |
| *GADD45B* | -0.6479 | 0.0321 |
| *MAEA* | -0.4781 | 0.0321 |
| *SYAP1* | -0.5436 | 0.0321 |
| *NDST2* | -0.6510 | 0.0321 |
| *RSBN1* | -0.5756 | 0.0321 |
| *CYTSA* | -0.7388 | 0.0321 |
| *ETS2* | -0.5919 | 0.0321 |
| *SLC9A8* | -0.3946 | 0.0321 |
| *ERLIN1* | -0.7029 | 0.0321 |
| *CHMP4B* | -0.3051 | 0.0321 |
| *SEC16A* | -0.4682 | 0.0321 |
| *ARFGAP3* | -0.5000 | 0.0321 |
| *DCUN1D2* | -0.2748 | 0.0321 |
| *LAT2* | -0.3765 | 0.0321 |
| *BMX* | -0.8410 | 0.0321 |
| **Gene Symbol** | **Log2 FC** | **adj.P.Val** |
| *BIN3* | -0.3605 | 0.0321 |
| *PLK3* | -0.5121 | 0.0321 |
| *CPD* | -0.6439 | 0.0334 |
| *JOSD1* | -0.5162 | 0.0344 |
| *ABCC4* | 0.3138 | 0.0344 |
| *CCNL1* | -0.4785 | 0.0347 |
| *RBMXL1* | -0.5569 | 0.0360 |
| *IQSEC1* | -0.5613 | 0.0360 |
| *KDM3B* | -0.4899 | 0.0365 |
| *G0S2* | -1.0984 | 0.0375 |
| *MLLT1* | -0.6059 | 0.0375 |
| *---* | -0.3056 | 0.0375 |
| *REL* | -0.5187 | 0.0375 |
| *KAT5* | -0.3363 | 0.0375 |
| *MON1B* | -0.4608 | 0.0382 |
| *FRMD4B* | -0.9117 | 0.0389 |
| *SERPINB9* | -0.6323 | 0.0394 |
| *GPR97* | -0.5520 | 0.0394 |
| *ZDHHC5* | -0.2598 | 0.0394 |
| *CDADC1* | -0.8944 | 0.0394 |
| **Gene Symbol** | **Log2 FC** | **adj.P.Val** |
| *OSBPL9* | -0.5058 | 0.0394 |
| *LOC154761* | -0.6516 | 0.0394 |
| *GOSR1* | -0.2762 | 0.0408 |
| *PHF23* | -0.3153 | 0.0416 |
| *CHD7* | -0.5351 | 0.0421 |
| *NLRP3* | -0.5859 | 0.0421 |
| *PTP4A1* | -0.7965 | 0.0423 |
| *SBNO2* | -0.4103 | 0.0423 |
| *RHBDD2* | -0.4993 | 0.0435 |
| *ZNF445* | -0.3348 | 0.0448 |
| *PTPRE* | -0.5751 | 0.0451 |
| *SLA* | -0.6211 | 0.0451 |
| *PHACTR1* | -0.5162 | 0.0455 |
| *KDM5C* | -0.3620 | 0.0457 |
| *GRK6* | -0.4022 | 0.0462 |
| *METRNL* | -0.5205 | 0.0471 |
| *LIMK2* | -0.4548 | 0.0495 |
| *TSEN34* | -0.4858 | 0.0495 |
| *IVNS1ABP* | -0.5687 | 0.0495 |

For every gene, the lof2 fold change (FC) and multiple-test adjusted Benjamini-Hochberg P value (adj.P.Val) is depicted.
