# Supplementary material for: Association between delay in intensive care unit admission and the host response in patients with community-acquired pneumonia
Source: Ann Intensive Care. 2021 Sep 28;11:142. doi: 10.1186/s13613-021-00930-5 (PMC8478267; doi:10.1186/s13613-021-00930-5)
Supplement: Supplementary file 5 — Additional file 5: Figure S1. Common transcriptional response in blood leukocytes obtained on admission in patients with direct or delayed ICU-admission for community-acquired pneumonia relative to health. [file 13613_2021_930_MOESM5_ESM.docx]

***Figure E1. Common transcriptional response in blood leukocytes obtained on admission in patients with direct or delayed ICU admission for community-acquired pneumonia relative to health***

**
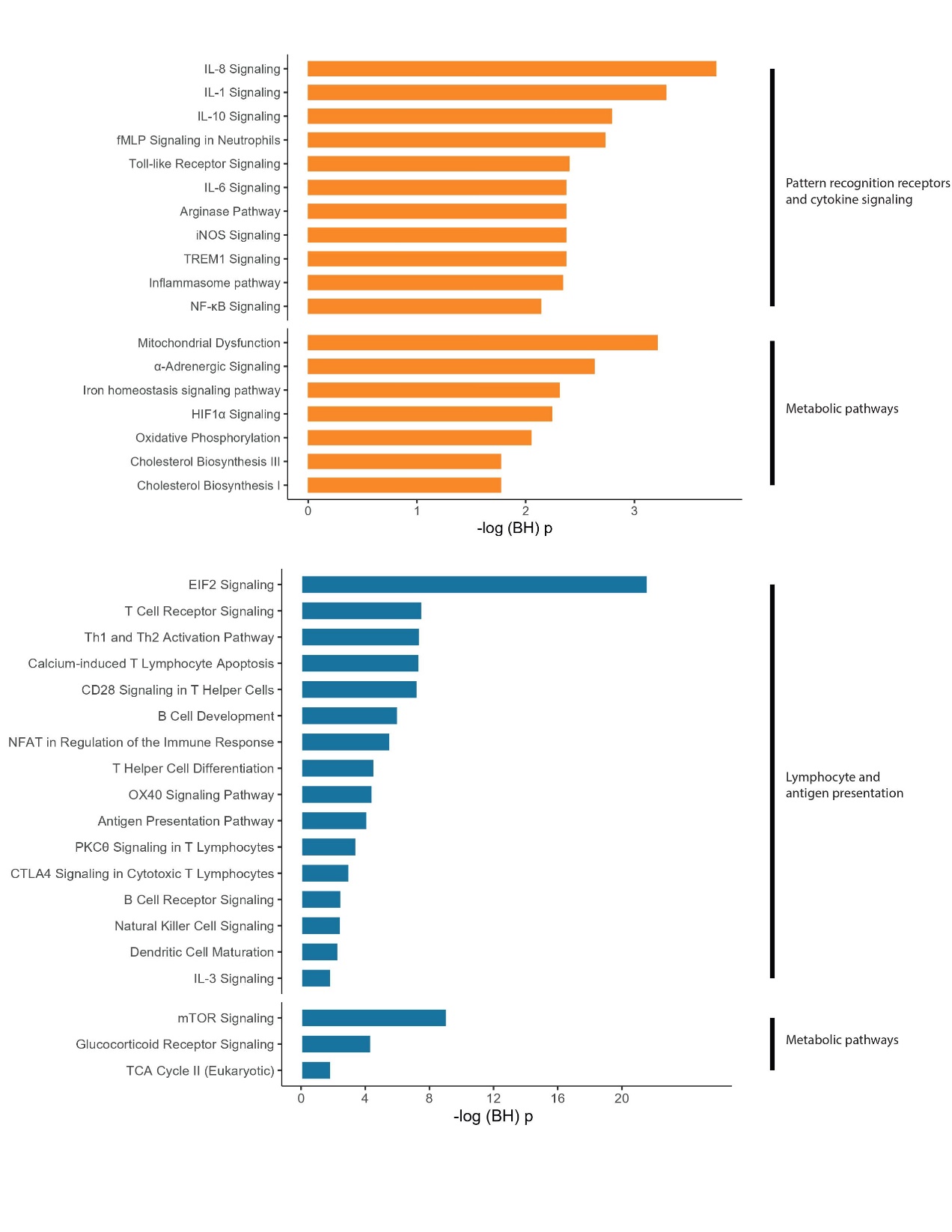
**

Considering Benjamini-Hochberg’s adjusted P<.05, over-expressed (orange, top), and under-expressed (blue, bottom) genes were analysed for association with canonical signalling pathways by Ingenuity pathway analysis (IPA, www.ingenuity.com). Significance was gauged by BH-adjusted Fisher exact probability. –log (BH) P, negative log transformed BH-adjusted P value
