# Supplementary material for: Association between delay in intensive care unit admission and the host response in patients with community-acquired pneumonia
Source: Ann Intensive Care. 2021 Sep 28;11:142. doi: 10.1186/s13613-021-00930-5 (PMC8478267; doi:10.1186/s13613-021-00930-5)
Supplement: Supplementary file 6 — Additional file 6: Figure S2. Leukocyte genomic responses in patients with community-acquired pneumonia with direct or delayed admission to the intensive care unit, all immunocompromised patients excluded. [file 13613_2021_930_MOESM6_ESM.docx]

***Figure E2. Leukocyte genomic responses in patients with community-acquired pneumonia with direct or delayed admission to the intensive care unit, all immunocompromised patients excluded***


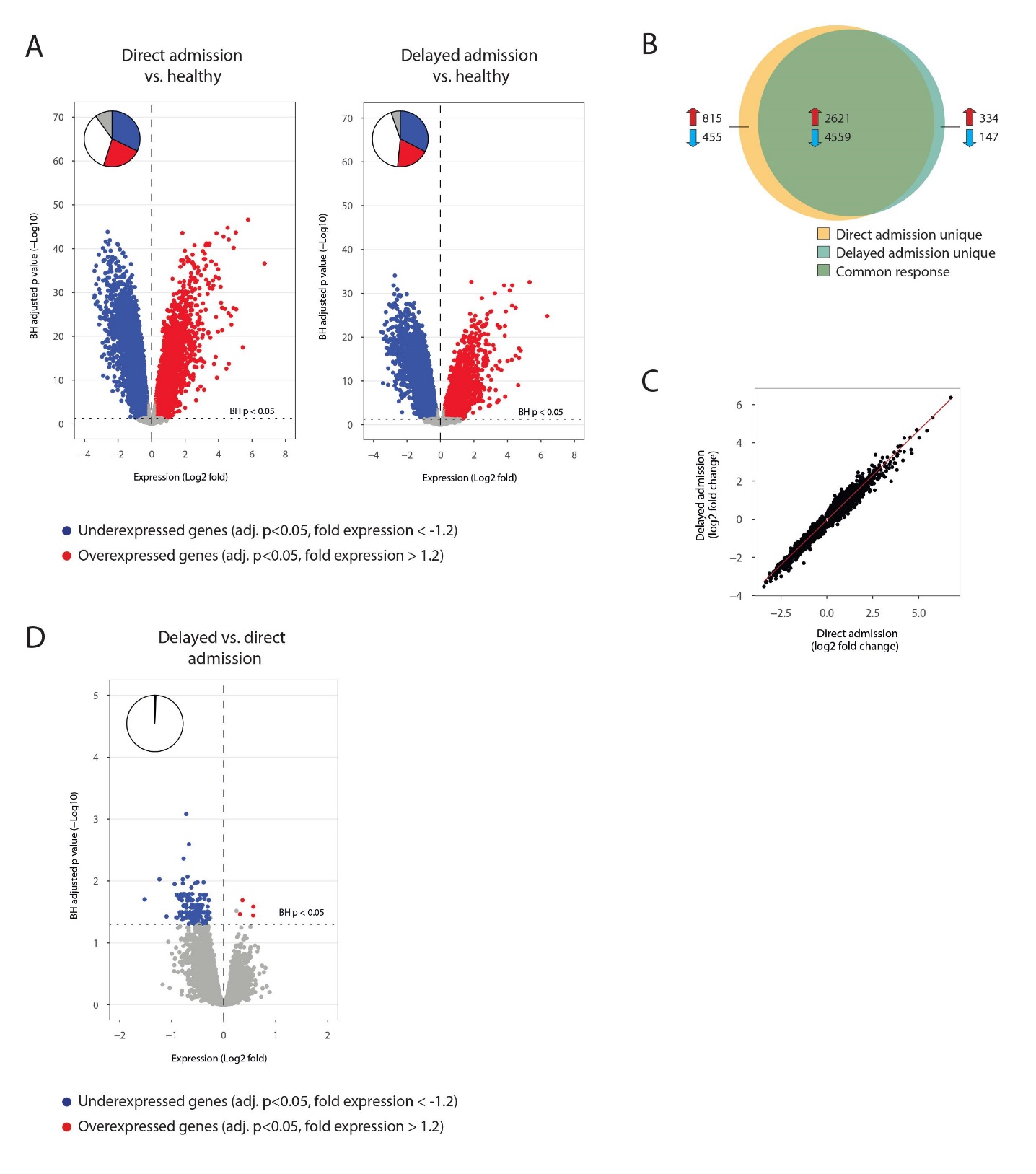


(A) Volcano plots illustrating the differences in leukocyte genomic responses (integrating log2 fold changes and multiple-test adjusted probabilities) between patients with direct admission to the ICU for community-acquired pneumonia (CAP) and healthy subjects (left), and patients with delayed admission for CAP and healthy subjects (right). Considering adjusted P<.05, 8450 and 7688 genes were identified as differentially expressed in patients admitted directly of with delay for CAP vs healthy subjects, respectively. Blue dots represent significantly underexpressed genes (adjusted P<.05, fold expression <-1.2) whereas red dots represent significantly overexpressed genes (adjusted P<.05, fold expression >1.2) in patients relative to healthy controls. Horizontal dotted line indicates multiple-test adjusted Benjamini-Hochberg (BH) P<.05 threshold. Within plots, pie charts show the extent of gene expression changes: blue slices show significantly underexpressed genes (adjusted P<.05 and expression more than 1.2-times decreased compared with healthy controls), red slices show significantly overexpressed genes (adjusted P<.05 and expression more than 1.2-time increased compared with healthy controls), and grey slices show significantly different gene expression (adjusted P<.05 and expression less than 1.2-time increased or decreased compared with healthy controls). (B) Venn-Euler representation of differentially expressed genes on admission in CAP patients with direct or delayed ICU-admission vs healthy subjects (adjusted P<.05). Red arrows denote overexpressed genes, blue arrows denote underexpressed genes. (C) Dot plot depicting the common response (log2 fold changes) of CAP patients with direct or delayed ICU-admission as compared with healthy subjects. Rho, Spearman’s correlation coefficient. (D) Volcano plot illustrating the differences in leukocyte genomic responses on admission between patients with delayed compared with direct admission to the ICU for community-acquired pneumonia (CAP). Considering adjusted P<.05, 111 genes were differentially expressed. –log (BH) P, negative log transformed BH-adjusted P value. Within plots, pie charts show the extent of gene expression changes in delayed compared to direct admissions for pneumonia.
